# Association of Physical Capacities with Peak Match Intensities and Match-to-Match Variability: An Exploratory Study in Young Male Football Players

**DOI:** 10.3390/sports14090371

**Published:** 2026-09-01

**Authors:** Marcus Lee, Sandave Singh, Muhammad Yusof, Vincenzo Rago, Frankie Tan

**Affiliations:** 1 Sport Science & Sport Medicine, UTR!, High Performance Sport Institute, Sport Excellence Singapore, Singapore 397630, Singapore; sandave_singh@hpsi.spexsg.org (S.S.); muhammad_sufian@hpsi.spexsg.org (M.Y.); vincenzo_rago@hpsi.spexsg.org (V.R.); 2 College of Sports Science, University of Kalba, Sharjah P.O. Box 11115, United Arab Emirates; 3 Sport Science & Sport Medicine, High Performance Sport Institute, Sport Excellence Singapore, Singapore 397630, Singapore; frankie_tan@sport.gov.sg; 4 Department of Physiology, Yong Loo Lin School of Medicine, National University of Singapore, Singapore 117593, Singapore

**Keywords:** physical capacity, peak intensity, power law, match variability, youth football

## Abstract

This study aimed to (i) describe the peak match intensities of youth football matches, (ii) quantify the variability of these peak intensities to provide references for monitoring players, and (iii) explore the association between physical capacities and peak match intensities. Match-running intensities were tracked for 32 young male footballers (age, 14.1 ± 0.8 y; height, 162.8 ± 8.3 cm; and 50.4 ± 9.6 kg) from three teams across 12 competitive matches. Peak match intensities were determined using a moving average technique, applied to total distance, high-speed distance (>14.4 km·h^−1^), average acceleration, and PlayerLoad™. The power-law relationships were constructed to derive an intercept and slope for the match variables, and linear mixed models were used to assess the association with physical capacities and estimate the between-player, between-match, and within-player variability. No clear associations were observed between any physical capacity and the intercepts or slopes derived from match-running intensities, with wide confidence intervals overlapping trivial effects. The coefficient of variation for peak match intensities was 7.1–20.9% (between-player), 4.4–10.3% (between-match), and 6.5–21.8% (within-player). The lack of a clear association between physical capacities and peak match intensities suggests that firm conclusions cannot be drawn from these findings. The reported peak intensities and match variability may aid performance practitioners to prescribe match-specific conditioning and monitor training drills but are specific to this cohort and competitive standard.

## 1. Introduction

Training in youth football involves the development of physical abilities to prepare players for competition. Part of this physical preparation involves competing in matches, characterized by periods of high-intensity efforts that impose substantial metabolic and neuromuscular demands [1]. Training studies in youth football have shown that match load typically forms the most demanding session within a week, reflecting that of professional football [2,3]. Thus, information derived from match load is crucial for optimizing training in youth football. During a football match, players are required to compete at short, peak intensity periods [4,5]. These periods often occur during fast transition phases influencing tactical behaviors at both individual and team levels, which may impact team performance [6].

To capture these peak intensity periods, a moving average of selected match parameters across different epochs can be applied to determine peak intensities. The peak intensities across various durations can then be derived from a power-law relationship that is fitted by plotting the log–log relationship of time on the *x*-axis and the intensities on the *y*-axis to derive the intercept and slope [7]. A power-law approach enables a continuous profiling of match-specific exercise intensities across durations—facilitating training prescriptions that estimate intensities for any given period, such as a 30 s interval. Establishing these peak intensities across continuous time periods subsequently allows for the prescription of training that is guided by the specific physiological and mechanical stressors encountered during a competitive match. Further, power-law regression values have demonstrated strong goodness of fit (R^2^ = 0.96–0.99) across GPS-derived variables related to speed, mechanical, and metabolic demands [8]. Power-law relationships have been used in both youth [5,9] and professional football [7,10], hockey [11], and rugby [8] to model peak match intensities across various durations. To our knowledge, limited evidence is available from Asian populations, with studies of peak match demands in youth football largely derived from Western populations [1,5,9,12]. Direct generalization of these findings to the physical preparation of school-based players may therefore be limited [13].

To effectively monitor players during training, it is also pertinent to examine the variability of the measurables. Understanding the variability in peak intensities between and within players provides a practical approach for interpreting match-to-match and player-to-player changes, ensuring that observed changes reflect meaningful or worthwhile alterations rather than typical match variations [14]. Previous studies have examined the variability of peak intensities across various playing standards, cohorts, and genders of football, where higher variability was shown in high-speed distance (coefficient of variation [CV]% = 7.6–34%), with relative distance and acceleration exhibiting lower variability (CV = 3.1–7.0%) [4,15]. However, the range of variability differs considerably across cohorts and standards of play, thus highlighting the necessity for quantifying between-match variation in peak running intensities within young football players.

In addition to establishing the duration–intensity relationship, it is also crucial to explore how individual physical capacities contribute to peak intensities. To date, studies in youth football examining the association between physical capacities and match-running output have focused primarily on the volume of match, revealing moderate-to-high associations (*r* = 0.56–0.70) between aerobic capacity and running output [16,17,18,19]. In rugby, studies that have examined peak intensities in relation to physical capacities [20,21] also revealed small-to-moderate associations (*r* = 0.46–0.60) of fitness capacities with the ability to attain and sustain peak running speeds and metabolic power. Conversely, the constructs of peak intensity and the rate of decline in intensity with duration may be conceptually closer to how physical capacities (sprint, jump, and intermittent aerobic capacity) are measured as compared to total match volume, which conflates high- and low-intensity phases of play. However, it is currently unknown if these associations exist in young football players.

With the scarcity of research in Asian youth football, particularly in the school context, an exploratory study is necessary to understand the unique characteristics of the cohort. The National School Games in Singapore is an annual inter-school competition that draws large participation of players, serving as a key platform in the development and identification of youth talents. Therefore, this study aimed to (i) describe the peak match intensities of youth football matches, (ii) quantify the variability of these peak intensities to provide references for monitoring changes between and within players, and (iii) explore the relationships between physical capacities and peak match intensities. Due to the exploratory nature of the study, no a priori hypotheses were established [22].

## 2. Methods

### 2.1. Study Overview

An observational design was employed to examine the relationship between peak intensities across incremental moving durations of 1–10 min and physical capacities. As this was an exploratory retrospective study, a formal a priori sample size calculation was not performed [22]. The sample size was determined by the data available. Data were collected across 12 matches (temperature = 31.1 ± 2.2 °C, humidity = 63.3 ± 9.4%, and heat index = 35.8 ± 4.4 °C) of the 2024 National School Games competition in Singapore. The adoption of an exploratory framework was informed by recent recommendations in sport and exercise science which advocate the use of such designs to discern patterns and associations within ecologically valid contexts [22]. This is particularly relevant in competitive settings involving under-researched player populations, such as Southeast Asian players.

For the assessment of physical capacities, the 40 m sprint test, vertical jump (VJ), and the Yo-Yo intermittent recovery level 1 (YYIRT1) were performed. All fitness tests were performed in the afternoon (temperature = 32.5 ± 1.7 °C, humidity = 61.8 ± 1.7%, and heat index = 34.0 ± 4.2 °C), conducted within four weeks of any matches played—a period we considered an acceptable representation of each player’s training status in relation to the proximity to the matches played [13].

### 2.2. Participants

Data were collected from 32 players across three teams (two U14 teams and one U16 team) over 12 matches, yielding 97 match observations. Pitch dimensions varied across matches, with a mean length of 90.8 ± 11.4 m and a mean width of 58.8 ± 6.8 m. Each team contributed between three and six matches (mean ± SD = 4.0 ± 1.7 matches), with players contributing between one and six matches (mean ± SD = 3.0 ± 1.5 matches). Only data from players who completed the first half of the match (U14, 35 min; U16, 40 min) were analyzed to limit the effect of pacing strategies, possible influence of substitution, and potential fatigue toward the end of the match [23]. Previous research has shown minimal differences in peak intensities between halves [24]. Goalkeepers were excluded from the analysis due to their different physical demands.

Physical capacities were measured once per player and paired with each player’s corresponding match observations, resulting in 32 player-level fitness profiles linked across the 97 match observations. The competitive level was Tier 2 according to McKay’s classification [25]. Ethical approval (SSI-IRB no. PH-EXE-054) was obtained from the Singapore Sport Institute. The participants’ anthropometric characteristics and physical capacities are summarized in Table 1.

### 2.3. Procedures

#### 2.3.1. Peak Match Intensity

Match data were collected using 10 Hz (GNSS) GPS units (Vector S7, firmware 8.5.1, Catapult Sports, Australia), which have previously shown good validity against reference tape measurements (bias in distance = 4.2 m) and reliability (CV = 0.17%) [26]. The inter-unit reliability was 0.5 to 2.0% CV across total distance, high-speed distance, and average acceleration [27]. All players wore the same units for each match. Data were downloaded using the manufacturer software (Openfield version 3.13, Catapult), with the raw velocity data extracted through the manufacturer’s API (CatapultR version 0.0.0.61, Catapult Sports) using a customized R script. The number of satellites received during data collection was 12.4 ± 0.4 (ideal range > 6), with a horizontal dilution of precision of 0.8 ± 0.1 (ideal range < 1.0). No match data were excluded from the analysis, as all recordings fell within these thresholds.

Four match-running performance metrics were used for the analysis of their peak match intensities: relative TD covered (m·min^−1^), relative HSR distance covered (>14.4 km·h^−1^), average acceleration (m·s^−2^), and PlayerLoad™ (au·min^−1^). The speed threshold of 14.4 km·h^−1^ was used as the HSR threshold, which was consistent with the academy’s standard classification, enabling comparison across age groups (U14 and U16) and benchmarking against the wider literature. This threshold reflects the lower end of high-speed running entry velocities reported in professional male soccer (14.4–21.1 km·h^−1^) [28]. Average acceleration was calculated through summing the absolute acceleration and deceleration speeds and averaging them over a defined time period to provide the overall total acceleration requirements of match-play [4]. PlayerLoad™ is derived from the manufacturer (Catapult). It is calculated as the sum of squared instantaneous changes in acceleration across three axes, divided by 100, representing overall mechanical ‘load’ [29]. The moving average for each metric was calculated for each player in 1 s increments across the entire first half, with the peak value for each duration (1–10 min) taken as the maximum across all window placements. For example, for each 1 min rolling average, the 600 consecutive data points (i.e., 10 samples per second for 60 s) with the highest average value; and a 2 min rolling average comprising 1200 data points, up to 10 min. Any 1 s interval without a valid data point was assigned a value of zero.

Individual player data for each match and metric were then used to construct a power-law curve to model the peak running intensities and durations [7]. To derive this relationship, a linear regression was fitted to the logarithm of intensity against the logarithm of time, yielding an intercept and slope specific to each curve. A predictive equation of running/mechanical load intensity (*i*) as a function of time (*t*) can therefore be expressed as *i* = c·*t*^n^, where c is the intercept, and n is the exponent describing the rate at which intensity declines as time increases.

#### 2.3.2. Physical Capacities

All tests were performed on artificial turfs between 3 and 6 pm (31.7 ± 2.1 °C, 66.5 ± 8.9% relative humidity, and heat index of 39.0 ± 4.2 °C). Before the field-testing session, anthropometric measurements (standing height and body mass) were taken for each participant. Thereafter, participants underwent a standardized warm-up (dynamic stretching, low-to-high-intensity running, jumps, and sprinting activities) led by a performance coach. Subsequently, the players were randomly allocated into two groups of approximately equal numbers to perform the 40 m sprint and vertical jump (VJ) tests. The Yo-Yo intermittent recovery test level 1 (YYIRT1) was performed as the last test of the session. The test–retest reliability of all tests was established previously in the academy with a smaller cohort of 11 players, 5 m (ICC = 0.76, CV = 2.6%), 40 m (ICC = 0.95, CV = 1.3%), MSS (ICC = 0.87, CV = 3.0%), VJ (ICC = 0.93, CV = 4.7%), and YYIRT1 (ICC = 0.72, CV = 12.8%). All reliability values were considered adequate.

##### 40 m Sprint

A 40 m sprint with 5, 20, and 30 m splits was performed to assess speed capacities (VALD SmartSpeed PRO, Newstead, Australia). Participants completed two warm-up runs at 70–80% effort, followed by two maximal-effort trials interspersed by 3-to-5 min rest between trials. The maximal sprinting speed (MSS) was calculated from the fastest running speed (lowest time) attained in either the 20–30 m or 30–40 m split [30].

##### Vertical Jump

For power capacity, vertical jump height was assessed with a yardstick incorporating arm swing [31]. Players practiced two warm-up jumps before attempting two maximal-effort jumps interspersed with ~ 10 s between trials. The maximum height of two attempts was used as the jump height score.

##### Yo-Yo Intermittent Recovery Test Level 1

The YYIRT1 was used to assess endurance capacity. The testing protocol consisted of two 20 m shuttle runs at increasing speeds, interspersed with a 10 s active recovery (controlled by audio beeps). The first failure to complete a shuttle resulted in a verbal warning, with the second failure resulting in the player being withdrawn. The total distance completed, including the shuttle of the failure, was recorded as the final test score [32].

### 2.4. Statistical Analysis

Peak match intensities derived from rolling time windows (1–10 min) are inherently interrelated, resulting in substantial collinearity between duration-specific metrics [15]. Hence, to examine the relationship between peak match intensity and physical capacities, separate linear mixed models (n = 8) were used to determine the effect of physical capacities on the intercept (log-transformed) and slope of the match intensities (lmerTest R package, ver. 3.1-3). The intercept represents the maximal intensity at 1 min, while the slope indicates the rate of decline in intensity. This approach aligns with established performance–duration modelling in exercise physiology and provides a parsimonious representation of a player’s peak intensity [33]. The models were built sequentially, with all physical capacities simultaneously entered as fixed effects, with random intercept for player. The 5 m and 40 m times were reverse-scored so that higher values represent better performance. Age group was included as a fixed effect to account for differences in physical and physiological development between the two cohorts, as biological maturation was not directly assessed, followed by a random intercept for match to account for multiple players playing the same match. Each addition significantly improved model fit, as reflected by a progressive reduction in AIC. Variance inflation factor (VIF) diagnostics on the final model revealed severe collinearity between 40 m sprint speed and MSS (r = 0.98; VIF = 76.5 and 57.5 respectively); therefore, 40 m sprint speed was removed from the final model. This reduced the VIF to below 2.5 for all remaining fixed effects. Standardized coefficient estimates are reported given the differing predictor scales. The full model output can be found in Supplementary File S1 (Tables S1 and S2). As a sensitivity analysis, all reduced models were also refitted after excluding players with fewer than two match observations (n = 25) (Supplementary Tables S3 and S4), and this did not materially change the results. The full sample was retained for the primary analysis. The *t* statistics from the model were converted to an effect size (ES) correlation with magnitude of effect interpreted as (0.10, trivial; 0.11–0.30, small; 0.31–0.50, moderate; 0.51–0.70, large; 0.71–0.90, very large; and >0.90, almost perfect) [34].

The random intercepts of the mixed model were subsequently used to estimate the between-player, between-match, and within-player variability. Between-match and within-player random-effect variances were pooled to represent the normal match-to-match variability expected for an individual player. Given the differing precision of the two pooled variance components, the effective degrees of freedom were calculated using the Welch–Satterthwaite equation. The pooled standard deviation was back-transformed to obtain values in raw units and as a percentage (CV), with 95% confidence limits calculated on the log scale using the pooled degrees of freedom and the appropriate value from the t-distribution.

The smallest worthwhile change (SWC) for each metric was used as a threshold to determine a meaningful change [35]. Here, we used the effect-size principle to define a moderate change (0.6 × between-player SD) [35]. A moderate effect (0.6) was selected because the cohort was classified as Tier 2, for which greater performance variability is expected than in senior elite athletes, where smaller and more stricter magnitude thresholds (0.2) are typically applied [25,36]. Practical change was calculated using the observed between-match variability (pooled between-match and within-player SD) + SWC, with probabilities set at ≥75% via the magnitude-based decision approach [36]. This is to provide a reference value that considers both between-match and within-player variability and a change that is meaningful to monitor an individual.

## 3. Results

### 3.1. Peak Match Intensity

**Figure 1:**
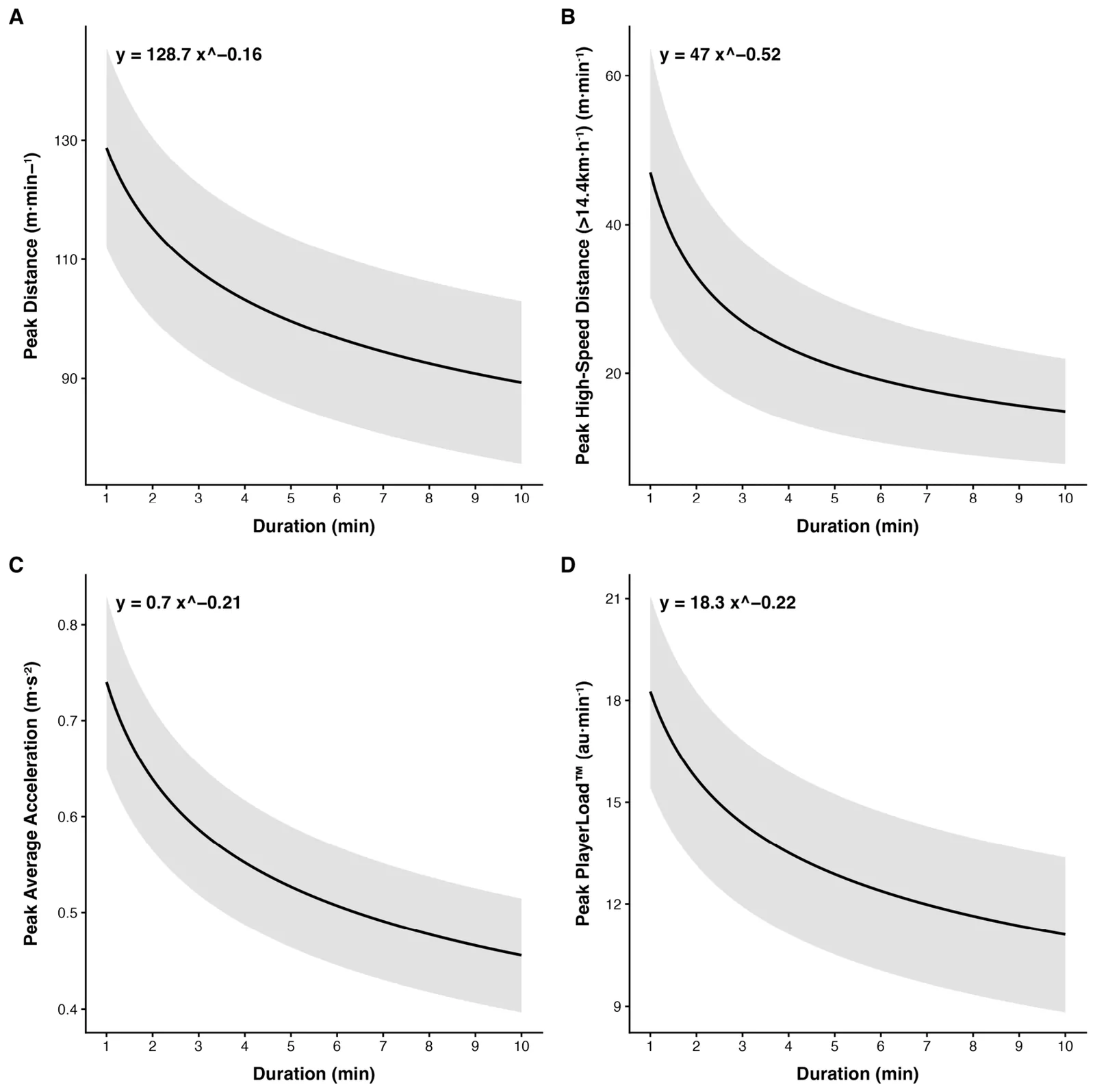
Power-Law relationship for peak duration-specific: (**A**) total distance, (**B**) high-speed distance, (**C**) average acceleration, and (**D**) PlayerLoad^TM^ (n = 32). The power-law equations were derived from the mean intercept and slope across all player-match curves; the black solid line and shaded band represent the pointwise mean and SD of individual curve predictions at each duration.

All power-law models constructed for each player across all match variables demonstrated large-to-perfect fits (Table 2). For average acceleration, three of the 97 player-match observations did not yield a complete data point across the 1–10 min duration range due to error in the raw 10 Hz GPS data, and were excluded from further analysis, resulting in a reduced sample of 94 curves for this metric. Peak distance, peak high-speed distance, peak average acceleration, and peak PlayerLoad™ followed a power-law relationship, indicating that running intensity per minute decreased as duration increased. The peak match intensities (power-law relationship) are illustrated in Figure 1.

### 3.2. Variability of Peak-Match Intensities

The variability in peak high-speed distance was the highest (between-player, 20.9%; between-match, 10.3%; and within-player, 21.8%), whereas variability was the lowest in peak total distance (between-player, 7.1%; between-match, 4.7%; and within-player, 6.7%), and peak average acceleration (between-player, 7.9%; between-match, 5.0%; and within-player, 6.5%). Full details of the variability are reported in Table 3.

### 3.3. Effect Size Correlation Between Physical Capacities and Most Intense Periods

**Figure 2:**
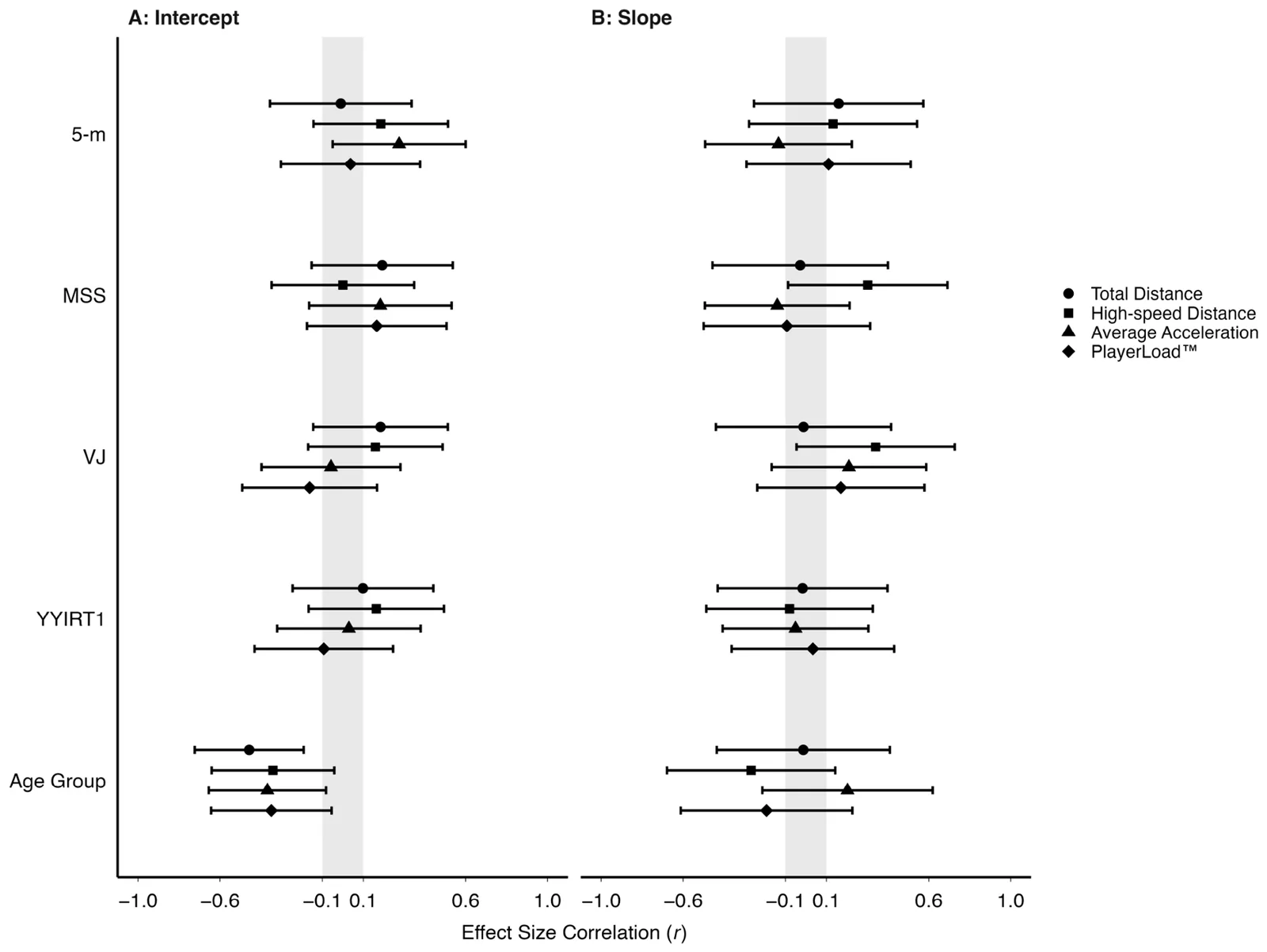
The association of fitness capacities and age group on the power-law intercepts and slopes of peak match intensity. Data (n = 32) are presented as effect size correlation with 95% confidence limits. The left panel (**A**) presents the association between the fixed effects and the intercept for each power-law intercepts and slopes. The right panel (**B**) presents the association between the fixed effects and the decline in maximum intensity measures as duration increases. The grey shaded area represents trivial correlation (−0.1–0.1); 5 m = 5 m sprint time (reverse scored); MSS = maximal sprint speed; VJ = vertical jump, YYIRT1 = Yo-Yo intermittent recovery test level 1.

A lack of clear association was observed between all the physical capacities and peak intensities (confidence limits overlap trivial magnitudes). Age group showed a small negative association (−0.45) with the intercept of total distance. The association of physical capacities with peak match intensities is illustrated in Figure 2.

## 4. Discussion

This study described (i) the peak match intensities of young male football players using power-law models; (ii) high between- and within-player, and between-match variability in peak running intensities, with variability increasing at higher running speeds; and (iii) unclear association between peak match intensities and physical capacities. The observed peak intensities for total distance, HSR, and acceleration were lower than those reported for elite youth footballers in older age groups, irrespective of the methodologies used for processing and deriving peak intensities [5,9,10,12]. These differences are likely attributed to different playing standards and age-groups, which impose distinct match demands across competitive levels. Specifically, higher playing standards have been shown to impose greater peak intensities, underscoring the need for tailored training prescriptions that reflect the specific physiological demands of each competition level and help avoid under- or overloading [10]. Importantly, these match-derived peak intensities incorporate change of direction and sport-specific actions (e.g., tackling and kicking) that are performed in multiple directions rather than being restricted to constant linear running. Thus, peak match intensities should not be used interchangeably with physiological intensity anchors, such as maximal aerobic speed, when prescribing run-based high-intensity interval training, as peak match intensities were lower than intensities derived from maximal aerobic speed [37].

The ability to monitor running-performance changes between matches and within players provides coaches with valuable insights into player management and exposure to match demand. In youth football, volume-based metrics (total distance, very high speed running, etc.) have shown wide variability (CV 3.8–57.4%), particularly in the HSR domain [14]. The peak match-running intensity (5 min) reported in young highly trained football players [14] was similar to the results observed in the present study. In contrast, our observed variability (within-player) in peak total distance was higher than that of professional football players [4,15], where lower CVs (~5–7%) for total distance have been reported. The within-player variability in very high-speed running (>19.8 km·h^−1^) in Novak et al. [15] was also comparable to the values reported in this study. Overall, this may be attributed to a range of contextual (e.g., match status and opposition quality) and tactical factors (e.g., possession phase and team formation) in match play that differ between cohort standards (youth vs. professional) [38]. The inherent limitations in the reliability of GPS measures during HSR would also contribute to the observed high variability, as these technologies can exhibit greater measurement error when running at higher velocities [26]. Further, the higher variability of peak total distance in the present study could also be explained by the sampling data (matches) assessed. Incorporating a greater number of matches would likely improve reliability, as demonstrated by comparisons of season-long versus short-term variability across match performance metrics, where shorter periods yield lower values [39]. The high variability of peak match intensities across various match performance metrics also reflects the multi-dimensional aspect of match intensities. With varied differences in variability, peak intensities would likely not manifest within a given similar time point [15]. This has implications for training and monitoring practices, where it may be useful to focus on a single metric for a given training exercise or drill based on the expected outcome or objective. Opportunities for HSR are structurally constrained in reduced pitch dimensions, making it unlikely that peak HSR and peak total distance would occur concurrently. Using the power-law-derived peak intensities, practitioners can prescribe sport-specific drill-based intensities replicating maximal intensity periods, thereby adequately exposing players to relevant training demands. For example, a SSG (4.0 min) to target overall movement and metabolic conditioning, relative total distance intensity is calculated as 128.7 × 4.0 − 0.16 = 103.1 m·min^−1^, yielding a total distance covered of 412.4 m. To monitor session-to-session, a difference of 10.4 m·min^−1^ from the expected intensity would highlight a practical change for the individual.

We acknowledge that pooling data from different age groups and teams to establish match variability may be a limitation, as it may introduce variability due to differences in team tactics and playing styles. Time intervals between matches may also contribute to variability in match-running performance which tend to be higher when data are collected in shorter time-frames (eight weeks) as opposed to the whole season [39]. However, all teams belonged to the same football academy and adhered to a guided game model and playing philosophy. Pooling across age groups reflected the academy’s practices at certain points in the season, when players from different age groups trained and competed together. Also, this may have value in bio-banded training or matches, with differing chronological age-groups training together [40]. Thus, understanding variability in mixed-age group settings remains valuable for practitioners in similar contexts. Future research should include a broader range of teams from varying performance levels within a given standard to enhance data heterogeneity and mitigate potential biases stemming from specific playing styles or training periodization.

The absence of an association between physical capacities and peak match intensities suggests that the relationship between fitness and peak match intensities remains inconclusive, as indicated by the wide and overlapping confidence intervals. This finding points to a more complex interplay of factors rather than a direct linear relationship. Previous research has identified associations between fitness and match performance in youth football players (*r* = 0.56–0.70) [17,18,19,41]. However, these studies have generally used fixed running speeds and aggregated running volume as outcome measures, rather than the peak intensities examined in the present study. Individualizing the high-speed running threshold may account for differences in relative fitness between players and may provide a more sensitive means of examining the relationship between peak intensities and physical capacities [42]. Notably, our findings using peak running intensity also differ from those observed in professional rugby players, where aerobic fitness (30–15 intermittent fitness test) and maximal squat strength (1-repetition maximum) were associated with peak distance covered (1–8 min) [21], and MSS showed a high association with peak speed and metabolic load [20]. The outcome differences used (peak intensities vs. volume) may explain divergent associations. Greater aerobic capacity (YYIRT1) was also associated with greater distances covered during the peak periods in female Australian football matches [43]. A plausible, though untested, explanation for this discrepancy may stem from the distinct physiological demands and tactical structures inherent to the sports, combined with the developmental stage of youth players who may not yet fully leverage their physical capacities in a consistent, maximal manner throughout a match. Moreover, the multifaceted nature of football, where running performance is largely a consequence of tactical play, opposition behavior, and evolving dynamics of the match itself may also explain the lack of association [6]. This inherent variability in match dynamics often necessitates strategic exertion, with players conserving energy for critical moments rather than consistently performing at their physiological limits [44]. This supports anchoring training intensity to peak, rather than average match intensities, so that players are better prepared for the stochastic pattern of high-intensity demands. Furthermore, as training drills are typically performed in short interval bouts, prescribing training intensity using peak match intensities is ecologically valid for coaches and practitioners.

Despite the inconclusive findings regarding the association between physical capacities and peak match intensities, continued fitness development remains essential for supporting young players’ pathway progression and preparing them to compete at higher competitive standards. Higher levels of physical capacity among players in the same age group competing in higher tiers of youth football have been consistently reported, highlighting the importance of developing these capacities to compete [45]. For example, possessing superior aerobic capacity may reduce overall physiological cost during intermittent high-intensity efforts and facilitates quicker inter-effort recovery, thereby enabling players to sustain high-intensity actions more frequently throughout a game without significant performance decrement [40]. Greater lower body strength has also been shown to reduce the magnitude of post-match neuromuscular fatigue in professional players [46]. This is especially important in the context of youth football tournaments, where congested matches are typically scheduled and necessitate recovery quickly after each match. However, as internal load, recovery status, and neuromuscular functions were not directly measured in the present study, these remain hypothesized rather than demonstrated mechanisms.

Interestingly, age group showed a small negative association with peak total distance, with the U14 age group recording a higher peak total distance than the U16 group. This finding warrants cautious interpretation. Age group reflects a chronological classification rather than biological maturation, which was not directly assessed in the present study. Therefore, the observed difference of age-group should not be interpreted as a maturational effect. With this caveat, chronological age may still relate to accumulated playing experience and tactical understanding of the game, independent of biological maturity. Younger players may tend to pace less and exert greater all-out effort because their tactical understanding is still developing. A previous study in young rugby players showed that when speed threshold was expressed relative to individual peak velocity, younger age group exhibited higher playing intensities [47]. Similarly, Thoseby et al. [10] also showed comparable peak total and high-speed distance between youth (U19) and senior players, although seniors exhibited higher peak average acceleration. Taken together, these findings suggest that peak demands may not differentiate competitive standard in a straightforward manner. Therefore, peak demands warrant closer examination to ensure that they can be appropriately translated into team-specific training prescriptions.

Several limitations should be considered when interpreting these findings. First, the wide and overlapping confidence intervals of the association between peak intensities and physical capacities should be interpreted as imprecise rather than as evidence of absence. Second, as age group showed a small effect on peak match intensities, direct assessment of biological maturation would be warranted, since biological age often exerts a greater influence on physical performance than chronological age during adolescence. Third, although the GPS units used have demonstrated good validity and reliability, match-to-match variability observed in high-speed running may have attenuated any true association between physical capacities and high-speed running intensity, contributing to the wide confidence intervals observed. Fourth, physical capacity testing was conducted within four weeks of the observed matches. While this window was considered an acceptable representation of each player’s training status, we cannot entirely rule out possible changes in fitness capacities. Lastly, the use of an absolute fixed threshold (>14.4 km·h^−1^) does not account for differences in high-intensity running, and may therefore represent different relative exercise intensities across players.

Future research should profile peak match intensities using single-age-group cohorts and incorporating internal load measures (e.g., heart rate), individualized running threshold, and contextual and tactical variables (e.g., possession, opposition standard, and match status) for various playing positions which may help clarify the physiological responses underlying peak match intensities. In addition, the dose–response relationship between training at peak match intensities could be explored to examine the impact of physiological adaption with training using peak match intensities periods.

## 5. Conclusions

In conclusion, this study served as an exploratory endeavor to document and understand peak match intensities in Tier 2 school-based youth football players competing in hot and humid conditions. Using the power-law approach, we described peak match intensities that may inform training prescription. Variability in peak match intensities differed across measures, with high-speed distance showing the highest variability. Collectively, these findings suggest that coaches and practitioners may use peak intensities as anchor points when prescribing and monitoring training drills. However, age group showed a small association with peak total distance, highlighting the need to consider age-related differences when prescribing training. The unclear associations between physical capacity and peak match intensities suggest that match-running demands are multifactorial. Future research should therefore examine these relationships further to improve the physical preparation of young football players for the demands of competition.

## Figures and Tables

**Table 1:** Summary of participants’ anthropometry and physical capacities.

Measures	Mean ± SD	Range
Age (y)	14.1 ± 0.8	13.4–15.5
Height (cm)	162.8 ± 8.3	139.4–176.5
Body Mass (kg)	50.4 ± 9.6	34.2–69.1
5 m (s)	1.08 ± 0.06	0.97–1.22
40 m (s)	5.80 ± 0.34	5.30–6.58
Maximal Sprinting Speed (km.h^−1^)	28.2 ± 2.1	24.2–31.3
Vertical Jump (cm)	49.4 ± 7.1	34.0–63.0
YoYo Intermittent Recovery Level 1 (m)	1013 ± 271	440–1640

**Table 2:** Power-law estimates for peak match intensities.

Measures	Curve (n)	Curves/Player (Mean; Range)	R^2^ (Mean ± SD)	Range	Intercept (Mean ± SD)	Slope (Mean ± SD)
TD	97	3.03 (1–6)	0.95 ± 0.05	0.80–1.00	128.7 ± 16.7	−0.16 ± 0.03
HSD	97	3.03 (1–6)	0.96 ± 0.02	0.90–1.00	47.0 ± 16.8	−0.52 ± 0.10
Ave Acc	94	3.00 (1–6)	0.95 ± 0.03	0.81–1.00	0.74 ± 0.09	−0.21 ± 0.04
PL	97	3.03 (1–6)	0.96 ± 0.03	0.86–1.00	18.3 ± 2.8	−0.22 ± 0.05

TD = total distance (m·min^−1^); HSD = high-speed distance (m·min^−1^) (>14.4 km·h^−1^), Ave Acc = average acceleration (m·s^−2^); PL = PlayerLoad™ (au·min^−1^).

**Table 3:** Variability of the peak-match intensities expressed in SD and CV (%).

Measures	Between-Player	Between-Match	Within-Player	Practical Change ^a,b^
**SD (raw units; 95% CI)**				
TD	5.6 [4.2, 7.1]	3.7 [2.0, 5.4]	5.3 [4.4, 6.2]	10.4 [8.5, 12.3]
HSD	20.7 [15.1, 26.5]	9.9 [5.2, 14.8]	21.6 [17.7, 25.8]	38.6 [31.9, 45.6]
Ave Acc	0.09 [0.06, 0.11]	0.05 [0.03, 0.08]	0.07 [0.06, 0.08]	0.14 [0.12, 0.17]
PL	1.9 [1.4, 2.3]	0.8 [0.4, 1.2]	1.6 [1.3, 1.9]	2.9 [2.4, 3.4]
**CV (%; 95% CI)**				
TD	7.1 [5.3, 8.9]	4.7 [2.5, 6.8]	6.7 [5.5, 7.9]	13.0 [10.7, 15.4]
HSD	20.9 [15.5, 26.3]	10.3 [5.5, 15.1]	21.8 [18.0, 25.6]	38.6 [32.3, 44.9]
Ave Acc	7.9 [5.8, 9.9]	5.0 [2.7, 7.4]	6.5 [5.3, 7.6]	13.1 [10.5, 15.7]
PL	10.1 [7.5, 12.8]	4.4 [2.3, 6.5]	8.8 [7.3, 10.4]	15.8 [13.2, 18.4]

^a^ Based on the combined between-match and within -player variability. ^b^ Practical change is calculated using the pooled between-match and within-player SD + smallest worthwhile change (SWC). TD = total distance (m·min^−1^), HSD = high-speed distance (m·min^−1^) (>14.4 km·h^−1^), Ave Acc = average acceleration (m·s^−2^), PL = PlayerLoad™ (au·min^−1^).

## Data Availability

Data are available from the corresponding author upon reasonable request. The R Code used for processing the catapult GPS data and the statistical analysis is available on request from the corresponding author.

## Supplementary Materials

The following supporting information can be downloaded at https://www.mdpi.com/article/10.3390/sports14090371/s1, Table S1: Linear mixed-model estimates of peak match intensity (intercept parameter) as a function of physical capacities and age group; Table S2: Linear mixed-model estimates of match-to-match decay rate (slope parameter) as a function of physical capacities and age group; Table S3: Sensitivity analysis: linear mixed-model estimates of peak match intensity (intercept parameter) as a function of physical capacities and age group, restricted to players with ≥2 match observations (n = 25 players); Table S4: Sensitivity analysis: linear mixed-model estimates of match-to-match decay rate (slope parameter) as a function of physical capacities and age group, restricted to players with ≥2 match observations (n = 25 players).

## References

[1] Doncaster G., Page R., White P., Svenson R., Twist C. (2020). Analysis of Physical Demands During Youth Soccer Match-Play: Considerations of Sampling Method and Epoch Length. Res. Q. Exerc. Sport.

[2] Hannon M.P., Coleman N.M., Parker L.J.F., McKeown J., Unnithan V.B., Close G.L., Drust B., Morton J.P. (2021). Seasonal Training and Match Load and Micro-Cycle Periodization in Male Premier League Academy Soccer Players. J. Sports Sci..

[3] Franceschi A., Robinson M.A., Owens D.J., Brownlee T., Bampouras T.M., Ferrari Bravo D., Enright K. (2024). Training Loads and Microcycle Periodisation in Italian Serie A Youth Soccer Players. J. Sports Sci..

[4] Thoseby B., Govus A.D., Clarke A.C., Middleton K.J., Dascombe B.J. (2022). Between-Match Variation of Peak Match Running Intensities in Elite Football. Biol. Sport.

[5] Kim S., Emmonds S., Bower P., Weaving D. (2023). External and Internal Maximal Intensity Periods of Elite Youth Male Soccer Matches. J. Sports Sci..

[6] Ju W., Doran D., Hawkins R., Gómez-Díaz A., Martin-Garcia A., Ade J., Laws A., Evans M., Bradley P. (2022). Contextualised Peak Periods of Play in English Premier League Matches. Biol. Sport.

[7] Delaney J.A., Thornton H.R., Rowell A.E., Dascombe B.J., Aughey R.J., Duthie G.M. (2018). Modelling the Decrement in Running Intensity within Professional Soccer Players. Sci. Med. Footb..

[8] Howe S.T., Aughey R.J., Hopkins W.G., Stewart A.M. (2022). Modeling Professional Rugby Union Peak Intensity–Duration Relationships Using a Power Law. Int. J. Sports Physiol. Perform..

[9] Duthie G.M., Thornton H.R., Delaney J.A., Connolly D.R., Serpiello F.R. (2018). Running Intensities in Elite Youth Soccer by Age and Position. J. Strength Cond. Res..

[10] Thoseby B., Govus A.D., Clarke A.C., Middleton K.J., Dascombe B.J. (2023). Peak Match Acceleration Demands Differentiate between Elite Youth and Professional Football Players. PLoS ONE.

[11] Delves R.I.M., Bahnisch J., Ball K., Duthie G.M. (2021). Quantifying Mean Peak Running Intensities in Elite Field Hockey. J. Strength Cond. Res..

[12] Jiménez S.L., Mateus N., Weldon A., Bustamante-Sánchez Á., Kelly A.L., Sampaio J. (2023). Analysis of the Most Demanding Passages of Play in Elite Youth Soccer: A Comparison between Congested and Non-Congested Fixture Schedules. Sci. Med. Footb..

[13] Rice J., Brownlee T.E., McRobert A.P., Ade J., Drust B., Malone J.J. (2022). The Association between Training Load and Physical Development in Professional Male Youth Soccer Players: A Systematic Review. Int. J. Sports Sci. Coach..

[14] Doncaster G., Unnithan V. (2019). Between-Game Variation of Physical Soccer Performance Measures in Highly Trained Youth Soccer Players. J. Strength Cond. Res..

[15] Novak A.R., Impellizzeri F.M., Trivedi A., Coutts A.J., McCall A. (2021). Analysis of the Worst-Case Scenarios in an Elite Football Team: Towards a Better Understanding and Application. J. Sports Sci..

[16] Buchheit M., Mendez-Villanueva A., Simpson B.M., Bourdon P.C. (2010). Match Running Performance and Fitness in Youth Soccer. Int. J. Sports Med..

[17] Rebelo A., Brito J., Seabra A., Oliveira J., Krustrup P. (2014). Physical Match Performance of Youth Football Players in Relation to Physical Capacity. Eur. J. Sport Sci..

[18] Bellistri G., Marzorati M., Sodero L., Sforza C., Bradley P.S., Porcelli S. (2017). Match Running Performance and Physical Capacity Profiles of U8 and U10 Soccer Players. Sport Sci. Health.

[19] Lovell T.W.J., Bocking C.J., Fransen J., Kempton T., Coutts A.J. (2018). Factors Affecting Physical Match Activity and Skill Involvement in Youth Soccer. Sci. Med. Footb..

[20] Duthie G.M., Thornton H.R., Delaney J.A., McMahon J.T., Benton D.T. (2020). Relationship Between Physical Performance Testing Results and Peak Running Intensity During Professional Rugby League Match Play. J. Strength Cond. Res..

[21] Glassbrook D.J., Fuller J.T., Wade J.A., Doyle T.L.A. (2022). Not All Physical Performance Tests Are Related to Early Season Match Running Performance in Professional Rugby League. J. Strength Cond. Res..

[22] Ditroilo M., Mesquida C., Abt G., Lakens D. (2025). Exploratory Research in Sport and Exercise Science: Perceptions, Challenges, and Recommendations. J. Sports Sci..

[23] Lacome M., Simpson B.M., Cholley Y., Lambert P., Buchheit M. (2018). Small-Sided Games in Elite Soccer: Does One Size Fit All?. Int. J. Sports Physiol. Perform..

[24] Thoseby B., Govus A.D., Clarke A.C., Middleton K.J., Dascombe B.J. (2023). Positional and Temporal Differences in Peak Match Running Demands of Elite Football. Biol. Sport.

[25] McKay A.K.A., Stellingwerff T., Smith E.S., Martin D.T., Mujika I., Goosey-Tolfrey V.L., Sheppard J., Burke L.M. (2022). Defining Training and Performance Caliber: A Participant Classification Framework. Int. J. Sports Physiol. Perform..

[26] Ocak M.F., Şentürk D., Akyildiz Z. (2025). Validity and Reliability of Catapult Vector S7 GNSS Units in Distance Measurement. BMC Sports Sci. Med. Rehabil..

[27] Crang Z.L., Duthie G., Cole M.H., Weakley J., Hewitt A., Johnston R.D. (2022). The Inter-Device Reliability of Global Navigation Satellite Systems during Team Sport Movement across Multiple Days. J. Sci. Med. Sport.

[28] Gualtieri A., Rampinini E., Dello Iacono A., Beato M. (2023). High-Speed Running and Sprinting in Professional Adult Soccer: Current Thresholds Definition, Match Demands and Training Strategies. A Systematic Review. Front. Sports Act. Living.

[29] Staunton C.A., Edholm P., Ide B.N.N., Ditroilo M., Wundersitz D. (2026). Playerload^TM^ and Accelerometer-Based Metrics: Scientific Evaluation and Implications for Athlete Monitoring. Front. Sports Act. Living.

[30] Buchheit M., Simpson B.M., Peltola E., Mendez-Villanueva A. (2012). Assessing Maximal Sprinting Speed in Highly Trained Young Soccer Players. Int. J. Sports Physiol. Perform..

[31] McLellan C.P., Lovell D.I., Gass G.C. (2011). The Role of Rate of Force Development on Vertical Jump Performance. J. Strength Cond. Res..

[32] Bangsbo J., Iaia F.M., Krustrup P. (2008). The Yo-Yo Intermittent Recovery Test: A Useful Tool for Evaluation of Physical Performance in Intermittent Sports. Sports Med..

[33] Drake J.P., Finke A., Ferguson R.A. (2024). Modelling Human Endurance: Power Laws vs Critical Power. Eur. J. Appl. Physiol..

[34] Rosnow R.L., Rosenthal R., Rubin D.B. (2000). Contrasts and Correlations in Effect-Size Estimation. Psychol. Sci..

[35] Hopkins W.G., Marshall S.W., Batterham A.M., Hanin J. (2009). Progressive Statistics for Studies in Sports Medicine and Exercise Science. Med. Sci. Sports Exerc..

[36] Hopkins W. (2017). A Spreadsheet for Monitoring an Individual’s Changes and Trend. Sportscience.

[37] Weaving D., Young D., Riboli A., Jones B., Coratella G. (2022). The Maximal Intensity Period: Rationalising Its Use in Team Sports Practice. Sports Med.-Open.

[38] Ju W., Morgans R., Webb J., Cost R., Oliva-Lozano J.M. (2025). Comparative Analysis of U17, U20, and Senior Football Team Performances in the FIFA World Cup: From Youth to Senior Level. Int. J. Sports Physiol. Perform..

[39] Gregson W., Drust B., Atkinson G., Salvo V. (2010). Match-to-Match Variability of High-Speed Activities in Premier League Soccer. Int. J. Sports Med..

[40] Abbott W., Williams S., Brickley G., Smeeton N. (2019). Effects of Bio-Banding upon Physical and Technical Performance during Soccer Competition: A Preliminary Analysis. Sports.

[41] Castagna C., Manzi V., Impellizzeri F., Weston M., Barbero Alvarez J.C. (2010). Relationship Between Endurance Field Tests and Match Performance in Young Soccer Players. J. Strength Cond. Res..

[42] Lee M., Mukherjee S. (2019). Relationship of Training Load with High-Intensity Running in Professional Soccer Players. Int. J. Sports Med..

[43] Black G.M., Gabbett T.J., Johnston R.D., Cole M.H., Naughton G., Dawson B. (2018). Physical Fitness and Peak Running Periods during Female Australian Football Match-Play. Sci. Med. Footb..

[44] Waldron M., Highton J. (2014). Fatigue and Pacing in High-Intensity Intermittent Team Sport: An Update. Sports Med..

[45] King M., Ball D., Gibson N.V., Weston M., Gallagher I.J., Dugdale J.H. (2026). Seasonal Physical Performance Changes in U12-U15 Male Youth Soccer Players. Int. J. Sports Med..

[46] Drayton A.M., Hamad M.J., Spyrou K. (2025). The Time Course of Postmatch Physical Impairments in Professional Soccer: A Systematic Review. J. Strength Cond. Res..

[47] Gabbett T.J. (2015). Use of Relative Speed Zones Increases the High-Speed Running Performed in Team Sport Match Play. J. Strength Cond. Res..

