# Supplementary material for: Association of Physical Capacities with Peak Match Intensities and Match-to-Match Variability: An Exploratory Study in Young Male Football Players
Source: Sports (Basel). 2026 Sep 1;14(9):371. doi: 10.3390/sports14090371 (PMC13611161; doi:10.3390/sports14090371)
Supplement: Supplementary file 1 [file sports-14-00371-s001.zip › sports-4435587-supplementary.pdf]

Supplementary Material

Table S1. Linear mixed-model estimates of peak match intensity (intercept parameter) as a function of physical capacities and age group.

Effect	Standardized β [95% CI]	df	t	p	Effect Size r [95% CI]
**Total Distance ($m \cdot min^{-1}$)**					
Intercept	0.63 [0.17, 1.09]	29.5	7.89	<0.001	0.82 [0.66, 0.91]
5-m	-0.01 [-0.35, 0.34]	30.5	-0.05	0.957	-0.01 [-0.35, 0.34]
MSS	0.20 [-0.17, 0.57]	28.7	1.05	0.302	0.19 [-0.18, 0.51]
VJ	0.18 [-0.16, 0.51]	31.8	1.06	0.298	0.18 [-0.17, 0.49]
YYIRT1	0.07 [-0.19, 0.33]	30.3	0.54	0.590	0.10 [-0.26, 0.43]
Age Group (U16 vs U14)	-1.22 [-2.03, -0.41]	33.9	-3.00	0.005	-0.46 [-0.68, -0.15]
**High-speed Distance ($m \cdot min^{-1}$) (>14.4 $km \cdot h^{-1}$)**					
Intercept	0.46 [0.00, 0.91]	30.6	2.75	0.010	0.45 [0.12, 0.69]
5-m	0.20 [-0.17, 0.58]	31.9	1.07	0.295	0.19 [-0.16, 0.49]
MSS	0.00 [-0.40, 0.41]	30.1	0.00	0.998	0.00 [-0.35, 0.35]
VJ	0.17 [-0.19, 0.52]	32.4	0.92	0.365	0.16 [-0.19, 0.47]
YYIRT1	0.13 [-0.15, 0.42]	31.9	0.94	0.356	0.16 [-0.19, 0.48]
Age Group (U16 vs U14)	-0.87 [-1.70, -0.04]	32.7	-2.08	0.046	-0.34 [-0.61, -0.01]
**Average Acceleration ($m \cdot s^{-2}$)**					
Intercept	0.57 [0.05, 1.09]	29.2	0.14	0.887	0.03 [-0.33, 0.38]
5-m	0.32 [-0.08, 0.71]	30.1	1.57	0.127	0.28 [-0.08, 0.57]
MSS	0.21 [-0.21, 0.64]	28.3	0.99	0.329	0.18 [-0.19, 0.51]
VJ	-0.06 [-0.45, 0.32]	31.6	-0.32	0.747	-0.06 [-0.39, 0.29]
YYIRT1	0.02 [-0.27, 0.32]	29.6	0.16	0.872	0.03 [-0.32, 0.38]
Age Group (U16 vs U14)	-1.10 [-2.04, -0.16]	34.4	-2.32	0.026	-0.37 [-0.62, -0.05]
**PlayerLoad™ ($au \cdot min^{-1}$)**					
Intercept	0.50 [0.03, 0.97]	30.5	3.77	<0.001	0.56 [0.27, 0.76]
5-m	0.04 [-0.35, 0.44]	31.5	0.21	0.834	0.04 [-0.31, 0.37]
MSS	0.20 [-0.23, 0.63]	29.9	0.92	0.365	0.17 [-0.19, 0.49]
VJ	-0.18 [-0.55, 0.20]	32.2	-0.93	0.358	-0.16 [-0.47, 0.19]
YYIRT1	-0.08 [-0.38, 0.22]	31.4	-0.52	0.605	-0.09 [-0.42, 0.26]
Age Group (U16 vs U14)	-0.95 [-1.82, -0.07]	33.5	-2.16	0.038	-0.35 [-0.61, -0.02]

β = standardized regression coefficient (predictors and outcome scaled to SD units); outcome modelled on the log scale. r = effect-size correlation derived from the t-statistic ($r = t/\sqrt{(t^2+df)}$), with 95% CI obtained via the Fisher z-transformation. Models included player identity and opposing team as random intercepts (1 | athlete) + (1 | opposition), estimated by maximum likelihood with degrees of freedom estimated via the Satterthwaite approximation. Based on 32 players and 12 opposing teams (97 player-match observations; 94 for average acceleration).

Table S2. Linear mixed-model estimates of match-to-match decay rate (slope parameter) as a function of physical capacities and age group.

Effect	Standardized β [95% CI]	df	t	p	Effect Size r [95% CI]
**Total Distance (m·min⁻¹)**					
Intercept	-0.00 [-0.52, 0.51]	18.5	-0.71	0.489	-0.16 [-0.56, 0.30]
5-m	0.11 [-0.19, 0.41]	19.9	0.72	0.478	0.16 [-0.28, 0.55]
MSS	-0.02 [-0.34, 0.30]	19.3	-0.12	0.904	-0.03 [-0.45, 0.41]
VJ	-0.01 [-0.30, 0.29]	19.4	-0.05	0.959	-0.01 [-0.44, 0.42]
YYIRT1	-0.01 [-0.24, 0.22]	20.7	-0.07	0.941	-0.02 [-0.43, 0.40]
Age Group (U16 vs U14)	-0.02 [-0.85, 0.80]	19.9	-0.06	0.955	-0.01 [-0.43, 0.41]
**High-speed Distance (m·min⁻¹) (>14.4 km·h⁻¹)**					
Intercept	0.20 [-0.25, 0.64]	19.1	-2.23	0.038	-0.45 [-0.74, -0.03]
5-m	0.09 [-0.20, 0.37]	20.5	0.61	0.552	0.13 [-0.30, 0.52]
MSS	0.22 [-0.09, 0.52]	20.0	1.42	0.172	0.30 [-0.14, 0.64]
VJ	0.22 [-0.05, 0.50]	19.4	1.59	0.127	0.34 [-0.10, 0.67]
YYIRT1	-0.04 [-0.26, 0.18]	21.4	-0.37	0.714	-0.08 [-0.47, 0.34]
Age Group (U16 vs U14)	-0.44 [-1.17, 0.29]	18.5	-1.20	0.247	-0.27 [-0.63, 0.19]
**Average Acceleration (m·s⁻²)**					
Intercept	-0.14 [-0.53, 0.24]	24.9	-1.53	0.139	-0.29 [-0.61, 0.10]
5-m	-0.13 [-0.48, 0.23]	27.4	-0.71	0.485	-0.13 [-0.47, 0.24]
MSS	-0.14 [-0.53, 0.24]	28.1	-0.75	0.459	-0.14 [-0.48, 0.23]
VJ	0.17 [-0.16, 0.50]	23.4	1.04	0.310	0.21 [-0.20, 0.56]
YYIRT1	-0.04 [-0.31, 0.23]	28.6	-0.27	0.787	-0.05 [-0.40, 0.31]
Age Group (U16 vs U14)	0.33 [-0.39, 1.06]	19.1	0.91	0.376	0.20 [-0.25, 0.58]
**PlayerLoad™ (au·min⁻¹)**					
Intercept	0.14 [-0.36, 0.64]	20.4	-0.98	0.337	-0.21 [-0.58, 0.23]
5-m	0.07 [-0.21, 0.36]	21.8	0.52	0.608	0.11 [-0.31, 0.49]
MSS	-0.06 [-0.36, 0.23]	21.3	-0.43	0.671	-0.09 [-0.48, 0.33]
VJ	0.11 [-0.17, 0.38]	20.3	0.78	0.444	0.17 [-0.27, 0.55]
YYIRT1	0.02 [-0.19, 0.23]	22.7	0.16	0.873	0.03 [-0.37, 0.43]
Age Group (U16 vs U14)	-0.34 [-1.13, 0.45]	18.9	-0.85	0.404	-0.19 [-0.58, 0.26]

Note. β = standardized regression coefficient (predictors and outcome scaled to SD units); the slope parameter was modelled in its native (untransformed) units. r = effect-size correlation derived from the t-statistic ($r = t/\sqrt{t^2+df}$), with 95% CI obtained via the Fisher z-transformation. Models included player identity and match as random intercepts (1|athlete) + (1|match), estimated by maximum likelihood with degrees of freedom estimated via the Satterthwaite approximation. Based on 32 players and 12 opposing teams (97 player-match observations; 94 for average acceleration).

Table S3. Sensitivity analysis: linear mixed-model estimates of peak match intensity (intercept parameter) as a function of physical capacities and age group, restricted to players with ≥2 match observations (n = 25 players).

Effect	Standardized β [95% CI]	df	t	p	Effect Size r [95% CI]
**Total Distance (m·min⁻¹)**					
Intercept	0.50 [0.01, 0.99]	27.75	1.98	0.058	0.35 [-0.01, 0.63]
5-m	-0.10 [-0.46, 0.25]	23.56	-0.57	0.574	-0.12 [-0.49, 0.29]
MSS	0.14 [-0.23, 0.50]	23.34	0.72	0.478	0.15 [-0.26, 0.51]
VJ	0.31 [-0.03, 0.65]	26.16	1.76	0.089	0.33 [-0.05, 0.62]
YYIRT1	0.22 [-0.06, 0.49]	22.84	1.55	0.135	0.31 [-0.10, 0.63]
Age Group (U16 vs U14)	-0.99 [-1.83, -0.16]	29.50	-2.34	0.027	-0.40 [-0.66, -0.05]
**High-speed Distance (m·min⁻¹) (>14.4 km·h⁻¹)**					
Intercept	0.30 [-0.20, 0.80]	27.60	1.17	0.251	0.22 [-0.16, 0.54]
5-m	0.05 [-0.34, 0.45]	24.47	0.27	0.789	0.05 [-0.34, 0.43]
MSS	-0.06 [-0.47, 0.36]	24.48	-0.27	0.792	-0.05 [-0.43, 0.34]
VJ	0.27 [-0.11, 0.64]	26.42	1.40	0.173	0.26 [-0.12, 0.58]
YYIRT1	0.28 [-0.03, 0.59]	23.90	1.78	0.088	0.34 [-0.05, 0.64]
Age Group (U16 vs U14)	-0.56 [-1.44, 0.31]	27.45	-1.26	0.219	-0.23 [-0.55, 0.14]
**Average Acceleration (m·s⁻²)**					
Intercept	0.35 [-0.19, 0.88]	26.99	1.28	0.212	0.24 [-0.14, 0.56]
5-m	0.15 [-0.23, 0.54]	23.89	0.78	0.440	0.16 [-0.24, 0.51]
MSS	0.14 [-0.26, 0.54]	23.66	0.68	0.504	0.14 [-0.27, 0.50]
VJ	0.12 [-0.25, 0.49]	26.57	0.62	0.542	0.12 [-0.26, 0.47]
YYIRT1	0.28 [-0.02, 0.57]	23.05	1.83	0.080	0.36 [-0.04, 0.66]
Age Group (U16 vs U14)	-0.71 [-1.63, 0.20]	29.54	-1.53	0.138	-0.27 [-0.57, 0.09]
**PlayerLoad™ (au·min⁻¹)**					
Intercept	0.43 [-0.09, 0.95]	29.04	1.61	0.119	0.29 [-0.08, 0.58]
5-m	-0.05 [-0.47, 0.37]	25.40	-0.24	0.812	-0.05 [-0.42, 0.34]
MSS	0.12 [-0.32, 0.56]	25.35	0.52	0.610	0.10 [-0.29, 0.46]
VJ	-0.04 [-0.44, 0.35]	27.26	-0.22	0.829	-0.04 [-0.40, 0.33]
YYIRT1	0.07 [-0.26, 0.40]	24.88	0.42	0.676	0.08 [-0.31, 0.45]
Age Group (U16 vs U14)	-0.85 [-1.77, 0.08]	28.32	-1.80	0.082	-0.32 [-0.61, 0.04]

β = standardized regression coefficient (predictors and outcome scaled to SD units); outcome modelled on the log scale. r = effect-size correlation derived from the t-statistic ($r = t/\sqrt{(t^2+df)}$), with 95% CI obtained via the Fisher z-transformation. Models included player identity and matches as random intercepts (1|athlete) + (1|match), estimated by maximum likelihood with degrees of freedom estimated via the Satterthwaite approximation. Sensitivity analysis excludes the 7 players (of 32) who contributed only a single match observation, retaining 25 players (90 player-match observations for total distance, high-speed distance and PlayerLoad™.

Table S4. Sensitivity analysis: linear mixed-model estimates of match-to-match decay rate (slope parameter) as a function of physical capacities and age group, restricted to players with ≥2 match observations (n = 25 players).

Effect	Standardized β [95% CI]	df	t	p	Effect Size r [95% CI]
**Total Distance (m·min⁻¹)**					
Intercept	-0.04 [-0.54, 0.46]	15.76	-0.16	0.874	-0.04 [-0.50, 0.44]
5-m	0.06 [-0.25, 0.36]	17.41	0.38	0.706	0.09 [-0.37, 0.52]
MSS	-0.09 [-0.41, 0.23]	18.13	-0.54	0.596	-0.13 [-0.54, 0.33]
VJ	0.05 [-0.24, 0.35]	17.02	0.36	0.726	0.09 [-0.38, 0.52]
YYIRT1	0.02 [-0.21, 0.25]	17.00	0.16	0.872	0.04 [-0.42, 0.49]
Age Group (U16 vs U14)	0.08 [-0.72, 0.88]	17.95	0.19	0.853	0.04 [-0.41, 0.48]
**High-speed Distance (m·min⁻¹) (>14.4 km·h⁻¹)**					
Intercept	0.12 [-0.33, 0.57]	15.37	0.51	0.617	0.13 [-0.37, 0.57]
5-m	0.07 [-0.21, 0.35]	19.44	0.49	0.627	0.11 [-0.33, 0.51]
MSS	0.21 [-0.09, 0.50]	20.24	1.37	0.187	0.29 [-0.15, 0.63]
VJ	0.24 [-0.03, 0.52]	18.76	1.73	0.100	0.37 [-0.08, 0.69]
YYIRT1	0.02 [-0.19, 0.24]	19.00	0.22	0.828	0.05 [-0.39, 0.47]
Age Group (U16 vs U14)	-0.26 [-0.99, 0.46]	17.98	-0.71	0.490	-0.16 [-0.57, 0.30]
**Average Acceleration (m·s⁻²)**					
Intercept	-0.17 [-0.58, 0.25]	14.08	-0.79	0.441	-0.21 [-0.64, 0.32]
5-m	-0.13 [-0.50, 0.25]	20.73	-0.66	0.517	-0.14 [-0.53, 0.29]
MSS	-0.14 [-0.53, 0.25]	22.67	-0.70	0.492	-0.15 [-0.51, 0.27]
VJ	0.13 [-0.20, 0.47]	18.56	0.78	0.447	0.18 [-0.28, 0.57]
YYIRT1	-0.04 [-0.33, 0.24]	20.57	-0.30	0.768	-0.07 [-0.47, 0.36]
Age Group (U16 vs U14)	0.38 [-0.39, 1.14]	15.85	0.97	0.348	0.24 [-0.26, 0.63]
**PlayerLoad™ (au·min⁻¹)**					
Intercept	0.12 [-0.37, 0.61]	14.84	0.47	0.644	0.12 [-0.38, 0.57]
5-m	0.05 [-0.24, 0.34]	18.86	0.33	0.748	0.07 [-0.37, 0.49]
MSS	-0.06 [-0.37, 0.24]	19.69	-0.40	0.696	-0.09 [-0.50, 0.35]
VJ	0.11 [-0.18, 0.39]	17.86	0.75	0.463	0.17 [-0.29, 0.57]
YYIRT1	0.04 [-0.18, 0.26]	18.47	0.34	0.734	0.08 [-0.37, 0.50]
Age Group (U16 vs U14)	-0.26 [-1.03, 0.52]	17.24	-0.65	0.525	-0.15 [-0.57, 0.32]

Note. *β = standardized regression coefficient (predictors and outcome scaled to SD units); the slope parameter was modelled in its native (untransformed) units prior to standardization. r = effect-size correlation derived from the t-statistic ($r = t/\sqrt{(t^2+df)}$), with 95% CI obtained via the Fisher z-transformation. Models included player identity and matches as random intercepts (1|athlete) + (1|match), estimated by maximum likelihood with degrees of freedom estimated via the Satterthwaite approximation. Sensitivity analysis excludes the 7 players (of 32) who contributed only a single match observation, retaining 25 players (90 player-match observations for total distance, high-speed distance and PlayerLoad™.*
